# Case Report: Spontaneous pneumomediastinum in a patient with MDA5-positive dermatomyositis and severe pulmonary fibrosis

**DOI:** 10.3389/fmed.2025.1646791

**Published:** 2025-10-13

**Authors:** M. Rizzo, C. Braga, M. Raschellà, B. Maranini, G. Schifino, A. Carnevale, M. Govoni, A. Lo Monaco

**Affiliations:** ^1^Rheumatology Unit, Department of Medical Sciences, University of Ferrara and Azienda Ospedaliero-Universitaria S.Anna, Ferrara, Italy; ^2^Respiratory Medicine Unit, Department of Cardio-Thoracic-Vascular Pathologies, Arcispedale Sant'Anna, Ferrara, Italy; ^3^Radiology Unit, Department of Translational Medicine, University of Ferrara, Ferrara, Italy

**Keywords:** case report, dermatomyositis, anti-MDA5 antibody, pulmonary fibrosis, spontaneous pneumomediastinum

## Abstract

**Introduction:**

Dermatomyositis (DM) is a rare autoimmune disorder, with the anti-melanoma differentiation-associated gene 5 (anti-MDA5) antibody positive subtype associated with severe complications such as rapidly progressive interstitial lung disease (RP-ILD) and, more rarely, spontaneous pneumomediastinum (SPM). This case highlights the challenges of managing a patient with such complex condition, particularly in the context of multiple comorbidities, including a history of cancer and recurrent infections.

**Case description:**

A 45-year-old woman with an history of vulvar squamous cell carcinoma (July 2022) presented with classic features of DM, including Gottron’s papules, proximal muscle weakness, dysphagia, severe cutaneous vasculitis and lymphopenia. She was then diagnosed with anti-MDA5-positive DM in October 2023. High-resolution CT (HRCT) of the lung performed in January 2024, in the absence of respiratory symptoms, revealed early interstitial changes with ground-glass opacities. Initial corticosteroid therapy yielded partial improvement. A *Listeria monocytogenes* meningitis in July 2024, coupled with her history of cancer, delayed the start of aggressive immunosuppressive therapy, even though the onset of dyspnea and imaging in June 2024 had already revealed worsening interstitial lung disease (ILD). In the meantime she received two cycles of intravenous immunoglobulin (IVIg). By October 2024, clinical decline with cutaneous ulcers and severe lymphopenia prompted cyclosporine (CyA) initiation. Concurrently, imaging revealed progressing ILD with new-onset pneumomediastinum and subcutaneous emphysema, culminating in massive emphysema requiring hospitalization and bilateral thoracic drainage. A multidisciplinary team approved the escalation to rituximab, which, in combination with her existing regimen, led to clinical stabilization and the resolution of her pulmonary and cutaneous symptoms.

**Conclusion:**

This case emphasizes the complexity of managing anti-MDA5-positive DM with severe pulmonary complications. Early recognition, a multidisciplinary approach, and personalized treatment are crucial to improving outcomes.

## Introduction

Dermatomyositis (DM) is a rare autoimmune disease classified among idiopathic inflammatory myopathies, characterized by proximal muscle weakness due to miopathy and distinctive skin rashes. It is commonly associated with specific autoantibodies, including the anti-melanoma differentiation-associated gene 5 (anti-MDA5) antibody. This particular subtype of DM often presents with skin manifestations, such as heliotrope rash, Gottron’s papules, and palmar papules, alongside systemic symptoms like muscle weakness and fatigue (1, 2) and is particularly notable for its association with rapidly progressive interstitial lung disease (RP-ILD), which significantly contributes to morbidity and mortality in affected patients (3–5). Spontaneous pneumomediastinum (SPM), defined as the presence of free air in the mediastinal space without an apparent cause, is a rare but serious complication observed in patients with DM, particularly those with anti-MDA5 antibodies. Based on large retrospective cohort studies, the incidence of SPM in patients with anti-MDA5-positive DM ranges from approximately 8.3 to 11.8% (6–10), while the prevalence is about 9–11% (10, 11) and its occurrence makes even more challenging the management of these patients (9, 12).

**Figure 1:**
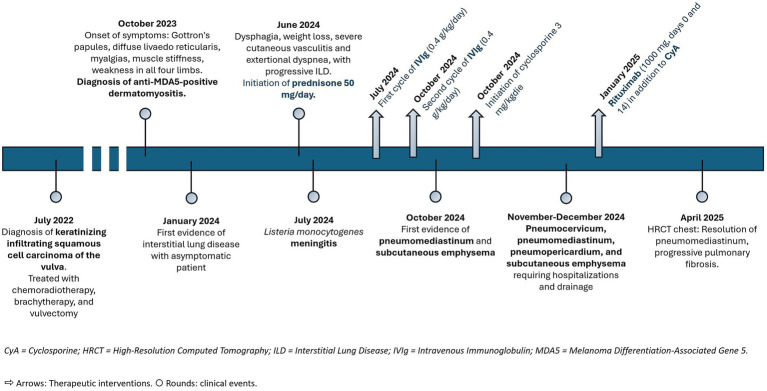
Diagram of disease progression, diagnostic evaluation and therapeutic interventions from July 2022 to April 2025. The timepoints indicate clinical events, while the arrows show therapeutic interventions.

Here, we present the case of a patient with anti-MDA5-positive DM who developed SPM in the context of advanced pulmonary fibrosis with an organizing pneumonia pattern. The clinical course was further complicated by cutaneous vasculitis, severe lymphopenia, and a history of vulvar squamous cell carcinoma and *Listeria monocytogenes* meningitis (Figure 1).

## Case description

A 45-year-old woman was diagnosed with anti-MDA5-positive DM after the onset of Gottron’s papules, diffuse livedo reticularis, myalgias, muscle stiffness and weakness in all four limbs in October 2023. Pulmonary involvement was first noted in January 2024, when the patient, asymptomatic for dyspnea and cough, underwent a high-resolution CT (HRCT) scan, which showed interlobular septal thickening and mild ground-glass opacities in the apical segments of both upper lobes.

**Figure 2:**
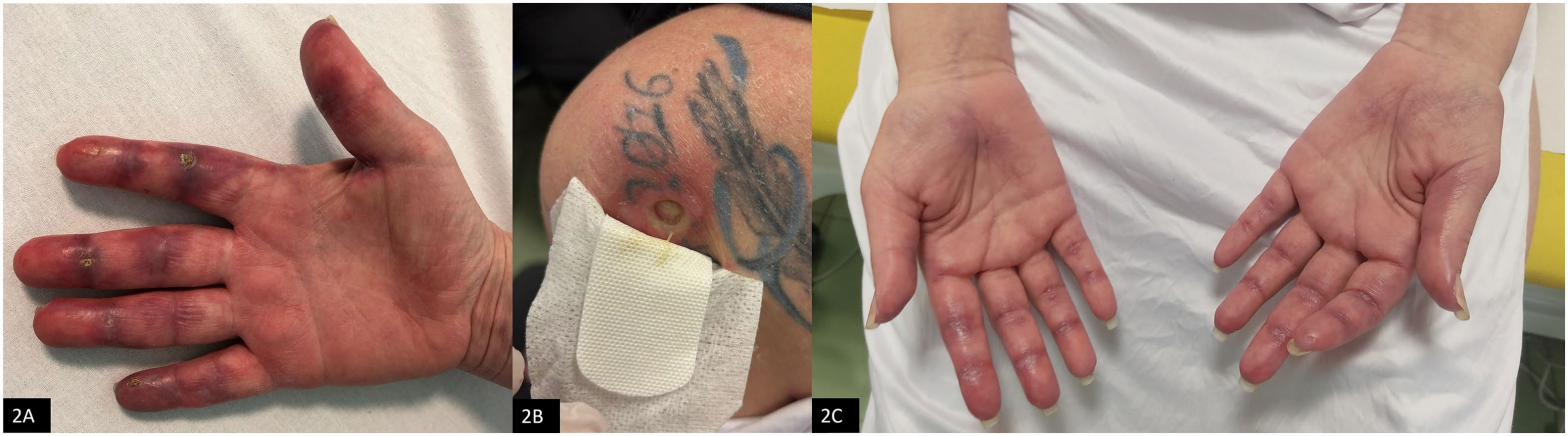
Cutaneous manifestations. **(A)** Cutaneous vasculitis of the hands at disease onset. **(B)** Cutaneous ulcer on the left supraspinous region, with a diameter of about 2 cm and surrounded by granulation tissue, in October 2024. **(C)** Skin involvement observed in April 2025, after treatment with rituximab plus Cyclosporine (CyA).

Upon admission to our Rheumatology Unit in June 2024 she also reported exertional dyspnea, dysphagia, weight loss, and presented severe cutaneous vasculitis (Figure 2A). Laboratory findings revealed positivity for anti-MDA5 and anti-Ro52 antibodies, while myocytolysis markers were within normal limits. Additionally, the patient exhibited marked lymphopenia, with 250 cells/μL (normal values 1,500–5,000 cells/μL). Her past medical history was significant for a diagnosis of vulva keratinizing infiltrating squamous cell carcinoma in July 2022, treated with chemoradiotherapy, brachytherapy and vulvectomy. Given her oncological background and persistent lymphopenia, immunosuppressive therapy was initially deferred pending oncological re-assessment. Instead, corticosteroid therapy was initiated (prednisone 50 mg/day), following a multidisciplinary discussion that highlighted a progressive ILD radiological pattern with organizing pneumonia (OP) at the follow-up HRCT performed during the hospitalization. Initially, glucocorticoid therapy led to partial clinical improvement; however, the therapeutic response waned over time.

In July 2024, she was hospitalized for bacterial meningitis caused by *Listeria monocytogenes*, which was treated with targeted antibiotics and followed by a prolonged rehabilitation program, without residual neurological sequelae.

Given the suboptimal response to corticosteroids, intravenous immunoglobulin (IVIg) therapy was started at a dose of 0.4 g/kg/day for 5 days at the end of July 2024. The treatment was well-tolerated and led to improvements in both cutaneous symptoms and dysphagia. Despite this initial success, the second cycle of IVIg administered in October 2024 did not result in significant benefits, with progression of skin lesions and the appereance of cutaneous ulcers (Figure 2B).

Due to persistent lymphopenia (up to 80 cells/μL) and heightened infectious risk, prophylactic therapy with cotrimoxazole (160 mg + 800 mg on alternate days) and aciclovir (400 mg twice daily) was initiated following consultation with infectious disease specialists. Considering the severity of her autoimmune disease and following multidisciplinary discussion with a haematologist, immunosuppressive therapy with cyclosporine (CyA) 3 mg/kg/die was started.

**Figure 3:**
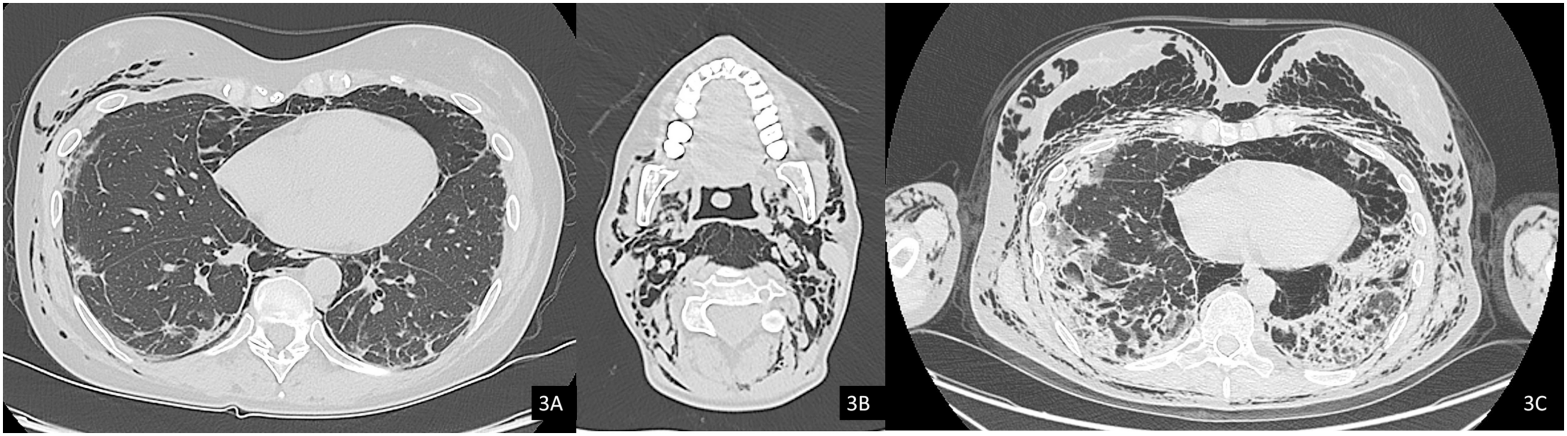
Axial computed tomography (CT) images, lung window. **(A)** Evidence of pneumomediastinum and interstitial emphysema with dissection through the extrapleural spaces and soft tissues of the chest wall. **(B)** Axial image at the cervical level showing pneumocervicum. **(C)** Subsequent chest CT scan showing marked worsening of subcutaneous and mediastinal emphysema.

In October 2024, a Positron Emission Tomography (PET) scan conducted as part of her routine oncologic surveillance revealed findings suggestive of interstitial lung disease (ILD), spontaneous pneumomediastinum, and subcutaneous emphysema, initially without evident signs on physical examination. Subsequently, the emphysema progressively developed, with rhinolalia and subcutaneous emphysema extending to the neck. An urgent CT scan of the neck and chest confirmed a diagnosis of pneumocervicum, pneumomediastinum, pneumopericardium, and subcutaneous emphysema (Figures 3A,B). Following a pulmonology evaluation, hospitalization was deemed necessary for further management. A conservative approach was adopted following thoracic surgery consultation, and low-flow oxygen therapy was initiated. A bronchoscopy was performed to obtain microbiological samples and to identify any pulmonary lesions that could have contributed to the air formation in the mediastinum. The procedure, however, was negative for significant findings, but was complicated by pulmonary consolidation, which was treated with piperacillin-tazobactam and linezolid. An esophagogastroduodenoscopy was also performed, which ruled out the presence of an esophageal fistula. After a follow-up CT scan that demonstrated improvement in the pneumomediastinum and pneumocervicum, the patient was discharged.

Subsequently, the patient was soon re-hospitalized as she experienced new episodes of acute rhinolalia and marked subcutaneous emphysema, extending from the chest to supraorbital region, with massive pneumomediastinum and pneumocervicum (Figure 3C). During this second hospitalization, two 28fr drainage segments were placed, resulting in significant air evacuation. The patient was then discharged on home oxygen therapy (2 L/min).

The case was discussed in a multidisciplinary team meeting, composed by pneumologists, radiologists, rheumatologists and thoracic surgeons. In light of the rapid radiological progression of ILD seen in serial chest CT scans, in the absence of macrofistulous tracts, the recurrent pneumomediastinum was attributed to severe pulmonary progression with diffuse fibrotic interstitial damage. Given the severity and rapid progression of the pulmonary disease, immunosuppressive therapy with rituximab (1,000 mg at day 0 and 14) was administered in January 2025, in addition to ongoing CyA.

After this combined treatment and interventional drainage, the patient achieved clinical stabilization, with no further recurrences of subcutaneous emphysema and progressive healing of skin manifestation (Figure 2C). Lymphocyte counts gradually increased, reaching values of 320/μL, and remaining stable.

**Figure 4:**
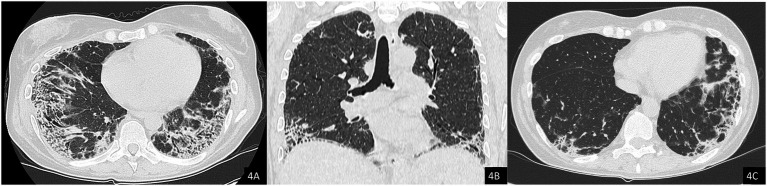
Axial **(A)** and coronal **(B)** chest CT images showing resolution of the pneumomediastinum and subcutaneous emphysema. Note the presence of interstitial lung disease (ILD) with a mixed NSIP/OP pattern and fibrosing features, including coarse reticulations, parenchymal distortion with traction bronchiectasis, and volume loss. **(C)** Comparison with a previous CT scan from 2024 demonstrates clear radiological progression of ILD.

The chest HRCT performed at the end of April 2025 (Figure 4) demonstrated complete resolution of the previously observed pneumomediastinum. When compared with the August 2024 scan (Figure 4C), imaging revealed progression of fibrotic interstitial changes. Although comparison with interim scans was limited by the presence of pneumomediastinum, there were no current radiological signs of active inflammatory disease.

## Discussion

SPM in anti-MDA5-positive DM is a rare complication, but its incidence is increasingly recognized, especially in patients with severe pulmonary fibrosis (7, 8). The incidence of pneumomediastinum in patients with anti-MDA5-positive DM has been reported to range from 8.3 to 11.8%, underscoring its clinical relevance and the need for heightened awareness among clinicians (3, 13). Notably, SPM can occur even in the absence of significant respiratory distress or specific radiological findings, thus complicating the clinical picture and requiring a high index of suspicion from healthcare providers (2, 14). The underlying pathophysiology of SPM in the context of DM remains poorly understood. Proposed mechanisms include the rupture of subpleural bullae or alveolar rupture secondary to ILD (14, 15). Alternatively, some authors have suggested that pneumomediastinum may result from underlying small-vessel vasculopathy. In fact, vascular abnormalities, such as cutaneous ulcers, a hallmark of this process, are frequently observed in affected patients (3, 4, 11, 16–18). In our case, the investigations performed ruled out the presence of macroscopic bullae or fistulae. Therefore, the pneumomediastinun was interpreted as being secondary be secondary to the underlying ILD disease or vasculopathy.

RP-ILD is another significant complication observed in our patient, which represents the most critical clinical manifestation of anti-MDA5-positive DM, affecting up to half of anti-MDA5-positive patients (19). This subgroup is often refractory to glucocorticoid and standard immunosuppressive therapies, resulting in a 6-month mortality rate as high as 50–70% (20).

From the onset, our patient displayed clinical and serological features indicating an elevated risk of severe complications. Firstly, the cutaneous presentation was extensive and severe from the outset, with the presence of deep skin ulcers, which are considered a significant prognostic marker in this subset of patients, strongly associated with respiratory complications, including spontaneous pneumomediastinum. This association likely arises from a shared underlying vascular pathology (2, 4, 12, 21).

Another parameter to consider is the severe and persistent lymphopenia observed in our patient, which is a typical finding of anti-MDA5-positive DM compared to other idiopathic inflammatory myopathies, and it serves as a marker of disease activity (22). Several studies have shown that lymphopenia is associated with an increased risk of developing pulmonary infections, rapidly progressive interstitial lung disease (RP-ILD), and spontaneous pneumomediastinum (9, 22).

Regarding pulmonary involvement, the patient also tested positive for anti-Ro52 antibodies, which are associated to an even more aggressive disease course, not only in DM but also in other idiopathic inflammatory myopathies (IIMs), such as anti-synthetase syndrome (23). Clinical studies have reported that individuals with both anti-MDA5 and anti-Ro52 antibodies experience more severe ILD, with faster progression of respiratory symptoms and a higher risk of mortality (24–29). This relationship emphasizes the importance of comprehensive serological testing in DM, as the identification of anti-Ro52 antibodies in MDA5-positive patients may help clinicians to predict disease trajectory and to tailor early aggressive immunosuppressive therapies accordingly. Overall, the presence of cutaneous ulcers, anti-Ro52 positivity, and lymphopenia may serve as prognostic indicators that support a more aggressive therapeutic approach.

This clinical case highlights the complexity in managing a patient with anti-MDA5-positive DM, not only due to the severity of the clinical presentation but also because of the significant comorbidities that limited therapeutic options.

The standard treatment for anti-MDA5-positive DM typically includes the use of aggressive immunosuppressants (30). Corticosteroids are frequently used to induce initial remission by controlling inflammation (12, 31). However, when corticosteroids alone are insufficient, other immunosuppressive agents such as mycophenolate mofetil, azathioprine, cyclophosphamide, and calcineurin inhibitors like cyclosporine and tacrolimus can be employed, as they have shown efficacy in the treatment of RP-ILD associated with DM (1, 3).

In our patient, the choice of immunosuppressive therapy was strongly influenced by her medical history of vulvar squamous cell carcinoma diagnosed approximately 1 year before the onset of DM. Coupled with the high risk of infection due to severe lymphopenia, the treatment approach had to balance the need for immunosuppression minimizing infectious risk. For this reason, the initial treatment was based solely on the administration of oral steroids at a medium-high dose (prednisone 50 mg/day with tapering), combined with two cycles of IVIg. Although definitive evidence regarding the role of IVIg in anti-MDA5-positive patients is still lacking, data from existing studies suggest potential benefits: IVIG therapy has demonstrated to be effective in a randomized controlled trial (RCT) involving patients with refractory DM, supporting its therapeutic potential (32). Current recommendations, such as those outlined by the British Society for Rheumatology, support a multimodal therapeutic approach in which IVIg may be considered in selected cases, particularly when the disease exhibits an aggressive course or when the patient presents contraindications to, or adverse reactions to other immunosuppressive agents (30).

Subsequently, due to the loss of efficacy of immunoglobulin therapy and persistent high disease activity, treatment with CyA was initiated at a dose of 3 mg/kg/day, in agreement with the haematologist. CyA, a calcineurin inhibitor, has been increasingly used as part of immunosuppressive regimens in patients with anti-MDA5–positive DM, particularly in cases presenting with RP-ILD (33). Several studies support the role of calcineurin inhibitors in controlling ILD in patients with DM and polymyositis (34, 35). The choice of CyA was also motivated by its non-lymphopenizing properties, a crucial consideration given the patient’s existing lymphopenia, and its use is also supported by British guidelines, which recommend CyA as an option for patients with rapidly progressive interstitial lung disease (30).

Finally, given the progressive complications of RP-ILD, spontaneous pneumomediastinum, and severe lymphopenia, a multidisciplinary approach was deemed necessary, leading to the decision to initiate rituximab (RTX) along with antiviral and antibiotic prophylaxis.

RTX is considered a second-line therapy when a combination of systemic corticosteroids and immunosuppressants fails (30, 33) and was chosen over cyclophosphamide (CYC) due to the known cytotoxic effect of CYC, which could have further worsened the lymphopenia, the patient’s history of infections and a previous oncological condition, as well as the presence of refractory cutaneous manifestations.

The use of RTX is supported by multiple guidelines. According to British Society guidelines, RTX is a recommended option for both refractory skin manifestations and for RP-ILD (30). Furthermore, the ACR guidelines consider RTX a first-line treatment for ILD in IIM (33). The rationale for its use is further supported by the Rituximab in Myositis (RIM) trial, the largest clinical trial conducted in IIMs (36). This trial demonstrated that 83% of both children and adults with DM, who had not responded to prior systemic corticosteroids and at least one immunosuppressant, improved with RTX and were able to reduce systemic steroid use more rapidly. Additionally, myositis-specific autoantibodies (MSA) positive individuals were more likely to respond favorably to RTX compared to MSA-negative individuals. In addition, there are several evidence regarding the use of RTX for pulmonary involvement. A systematic review by McPherson et al. (37) provides an overview of the management strategies adopted in anti-MDA5-positive clinically amyopathic DM-associated ILD, highlighting rituximab as a viable immunosuppressive option particularly in cases of refractory RP-ILD. Although much of the evidence primarily comes from case reports and observational studies rather than large randomized controlled trials, current evidence suggests that RTX may offer clinical benefits in managing pulmonary involvement, with an acceptable safety profile (36–40).

The multidisciplinary approach was crucial in managing the recurrent and progressive pneumomediastinum, which required the intervention of pulmonologists and thoracic surgeons (41). Massive subcutaneous emphysema, which involves extensive air trapping in the soft tissues, is a potentially life-threatening condition that can compromise both airway patency and cardiopulmonary stability. Although definitive guidelines are lacking, several case reports and case series in the literature support the use of subcutaneous drainage as an effective method for managing severe cases, facilitating the removal of trapped air, thereby reducing tissue tension and preventing further respiratory compromise (42–44).

The treatments received by the patient were consistently well tolerated, leading to both a subjective improvement and a slow but progressive clinical improvement, which included a partial recovery of her functional independence. She has since transitioned from being wheelchair-bound to being able to ambulate, and she can now perform most daily living activities on her own. She has also started a physical therapy program to support her ongoing recovery.

The prognosis for patients with pneumomediastinum associated with DM remains guarded, with studies indicating that the presence of this complication correlates with higher mortality rates (14, 21, 31). In our case, despite the resolution of the pneumomediastinum and the patient’s clinical stabilization, fibrotic progression of the underlying disease persisted. The partial yet favorable clinical response observed may open new perspectives on the potential use of antifibrotic therapy as a synergistic component of the ongoing therapeutic strategy for progressive pulmonary fibrosis (45).

Early recognition and prompt initiation of appropriate therapies are critical to improve outcomes. Ongoing research into the efficacy of various treatment modalities, including novel immunosuppressive agents and combination therapies (e.g., with anti-fibrotic agent), will be essential in refining management strategies for this complex and challenging condition.

## Conclusion

This case highlights the multifaceted challenges in managing a patient with anti-MDA5-positive DM. The patient’s history of cancer, along with multifaceted clinical and haematological concerns, further complicated the decision to initiate aggressive treatment, despite the severity of the clinical presentation. This forced the need for multidisciplinary decision-making, as close collaboration between specialties is crucial to providing comprehensive care, ensuring that each aspect of the patient’s condition is addressed in a timely and effectively.

Ultimately, this case underscores the urgent need for ongoing research to elucidate the pathophysiological mechanisms of anti-MDA5-positive MD and to establish optimal therapeutic strategies for managing pulmonary involvement in this condition.

## Data Availability

The original contributions presented in the study are included in the article/supplementary material, further inquiries can be directed to the corresponding authors.
